# Neurite guidance and neuro-caging on steps and grooves in 2.5 dimensions†

**DOI:** 10.1039/d0na00549e

**Published:** 2020-07-14

**Authors:** Cornelius Fendler, Jann Harberts, Lars Rafeldt, Gabriele Loers, Robert Zierold, Robert H. Blick

**Affiliations:** Center for Hybrid Nanostructures (CHyN), Universität Hamburg 22761 Hamburg Germany rzierold@physik.uni-hamburg.de; Center for Molecular Neurobiology Hamburg (ZMNH), University Medical Center Hamburg-Eppendorf (UKE) Hamburg 20251 Germany; Material Science and Engineering, College of Engineering, University of Wisconsin-Madison Madison Wisconsin 53706 USA

## Abstract

Directed guidance of neurites is a pre-requisite for tailor-made designs of interfaces between cells and semiconducting components. Grayscale lithography, reactive ion etching, and ultraviolet nanoimprint lithography are potent semiconductor industry-compatible techniques for a cost- and time-effective fabrication of modulated surfaces. In this work, neurite outgrowth of murine cerebellar neurons on 2.5D pathways produced with these methods is studied. Structures of micron-sized steps and grooves serve as cell culture platforms. The effects of contact guidance through topography and chemical guidance through selective poly-d-lysine coating on these platforms are analyzed. As a consequence, the herein presented fabrication approach can be utilized to cultivate and to study low-density neuronal networks in 2.5D configuration with a high degree of order.

## Abstract 2

Tailor-made structures of micron-sized steps and grooves serve as cell culture platforms to guide neurite outgrowth. The effects of contact guidance through topography and chemical guidance are analyzed.

## Graphical abstract

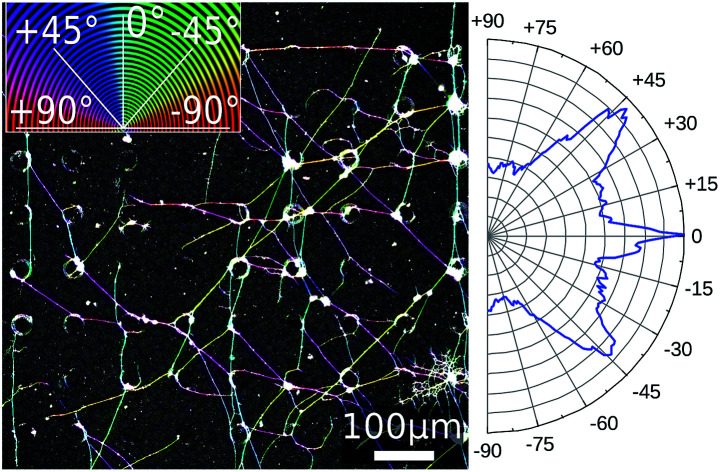

## Introduction

The ability to manipulate and direct axonal pathfinding is crucial for bioengineering defined neuronal circuits. Improved knowledge and control over axon outgrowth could lead to the development of diagnostics and cures for currently incurable and in part still poorly understood pathologies of the central nervous system such as Alzheimer's disease, multiple sclerosis or spinal cord injuries.^1^ Valuable tools to study neurons are *in vitro* cell cultures on micro-patterned cell culture platforms. Specifically, axon pathfinding and neuronal outgrowth can be controlled by chemical or topographical cues or a combination of them.^2^ A pioneering publication in the field of chemical guidance is the work of Kleinfeld *et al.* from 1988.^3^ They showed how micropatterning of planar substrates by photolithography utilizing di- and triamines as chemical binding centers promoted local cellular adhesion and outgrowth of rodent spinal and cerebellar cells whereas monoamines, such as (3-aminopropyl)triethoxysilane—which in fact are generally used as adhesion promoter to covalently bond organic materials to oxidic surfaces—inhibited the cell adhesion. Another approach was pursued by Oliva *et al.* which used microcontact printing to define arrays of a cell adhesion molecules serving as guide for the axon's outgrowth of hippocampal neuronal cells from embryonic rats but leaving the cell soma as well as dendrites unaffected.^4^ Note, both techniques employed to define purely chemical cues, namely photolithography and contact lithography, are standard tools in semiconductor and microelectronic industry for decades. On the other hand, topographical cues (partially in combination with chemically induced guidance) have been investigated to tailor neuronal outgrowth. A plethora of surface modulated or 2.5-dimensional (2.5D) structures have been utilized to act as culture platforms for guided neurite outgrowth, including variations in nanoscale surface topography,^5^ guidance barriers,^6,7^ 3D confinement in neuro-cages, grooves and channels,^8–15^ microtube arrays,^16–20^ nanowires,^21^ and nano- or micropillars.^22–26^ The synthesis of such tailor-made neurite guiding platforms has been motivated for their application in drug screening, scaffolding of artificial tissue, axon-specific testing of growth direction, modeling the myelin formation, and gaining insight into regeneration processes occurring after a severe injury of the spinal cord.^27–30^ Moreover, in recent years, brain-on-a-chip devices have aroused interest of the research community as pedant to organ-of-a-chip approaches. The latter ones not only focus on neuronal cells but also investigate other cells of the human body, such as liver, lung, or the skin in artificial devices.^31–33^

On the one hand, the progress in micro- and nanostructuring within the last decades allows for the synthesis of complex cell culturing substrates with tunnels and tubes being far beyond a simple surface structuring. As an example, arrays of self-rolled-up microtubes—upon release of intrinsic strain in their multilayer thin film architecture by selective etching of a sacrificial layer—or 3D-printed free-standing hollow channels by 2-photon-polymerization lithography can serve as artificial substrates for neurite guidance and confinement. However, the fabrication of complex surfaces geometries with the aforementioned procedures is time-consuming, expensive, not scalable, and hence economically not feasible for large-scale production as needed for *e.g.* drug screening experiments. Other techniques qualified for scalable applications, however, such as fibrinogen, cellulose nanofibrils, or hydrogels have been applied as cell culture substrates already but lack of structural features to manipulate neuronal outgrowth at will.^34–36^

On the other hand, substrates with tailor-made surface geometries for cell cultivation, can be prepared by making use of well-established methods in microelectronic industry, such as photolithography, microcontact printing, imprint lithography, dry- and wet-etching processes, physical and chemical vapor depositions methods to name a few of them. Moreover, such fabrication routes only make use of materials of synthetic nature and are thus free from ethical concerns and of general interest in the field.^37^ In future, ethical concerns could be further minimized by the application of induced stem cell-derived neurons which have been demonstrated to be cultivatable on microstructured substrates as well.^38,39^

In this work, we combine established technologies, namely grayscale lithography (GSL) and ultraviolet nano-imprint lithography (UV-NIL) with reactive ion etching (RIE) to prepare readily reproducible and cost-effective modulated surfaces. The topographical features of the platforms are specifically designed to study contact guidance during neuronal network formation. Cellular network orientation and neurite outgrowth pathways of murine cerebellar granular cells (MCGCs) through grooves and over micron-sized edges with varying heights are analyzed herein. In detail, lithographically structured steps inside of grooves introduce 2.5D modulation in a substrate to further enhance the complexity of the outgrowth pathways. Parylene C (ParC) and alumina (Al_2_O_3_) surface coatings are applied by chemical vapor deposition (CVD) and atomic layer deposition (ALD), respectively, before culturing for improved cell adhesion and viability.^40–47^ Furthermore, a spatially localized poly-d-lysine (PDL) coating is utilized to improve the selectivity of soma adhesion and neurite outgrowth paths.^3,48,49^ Moreover, additional laminin coating promotes neurite outgrowth and neuron viability.^50^

## Results and discussion

### Contact guidance at steps and in grooves

**Fig. 1:**
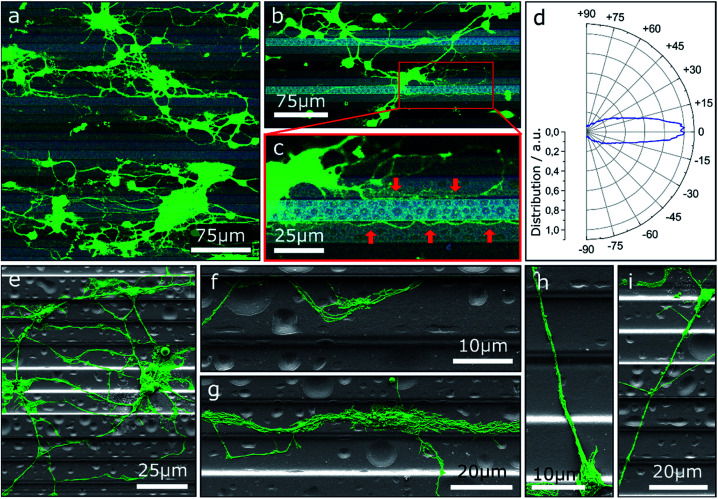
(a–c) CLSM images of MCGCs cultured on ParC coated step structures produced by GSL at 4 DIV. Brightness of the steps correlates with the height: light and dark correspond to top steps and bottom pits, respectively. Red arrows point to neurites guided along edges. (d) Circular histogram of the normed distribution of neurite orientations in (a) relative to the step edges. (e–i) False-colored scanning electron microscopy (SEM) images of neurons on step-structures after 4 DIV.

One approach to tailor the neuron outgrowth is to introduce morphological traces, which serve as guide for the growth cone. Fig. 1 shows neurons cultivated on step structures produced by GSL. The height difference between adjacent steps is 2 μm with a total of four different heights (see also Fig. S1†). Vital neuronal networks have developed on the substrates as displayed by confocal laser scanning microscopy (CLSM) images (Fig. 1a–c). A tendency to settle on lower levels on the structure rather than on the top steps is observable: 32% of the cells are seeded in the pits, 34% on the lower steps, 27% on the higher steps, and 8% on the top steps. The overall cell density on the displayed substrate is (648 ± 114) cells per cm^2^. Note, the pits and top steps each make up 1/6 of the total surface area, while lower and higher steps each make up 1/3. Consequently, the measured cell density is highest in the pits with (1086 ± 264) cells per cm^2^. Neuronal network formation over the entire substrate surface shows that the steps do not present an insurmountable barrier for neurite outgrowth. However, the outgrowth is significantly influenced by the topography and many neurites are oriented in the direction of the step edges. The majority of neurite trajectories is oriented within an angle of ±15° towards the step edge direction (Fig. 1d).

Further insight into the network geometry is provided by scanning electron microscopy (SEM) analysis of the same culture. Most neurites are oriented along the step edge direction for a large portion of their paths (Fig. 1e). Many thin neurites as well as neurite bundles with small impinging angles are guided and deflected by step edges (Fig. 1f and g). However, once steps are crossed, neurites are predominantly not deflected. In detail, the majority of observed neurites that cross multiple steps both upwards and downwards without deflection have a trajectory over 60° relative to the step edges (Fig. 1h and i).

**Fig. 2:**
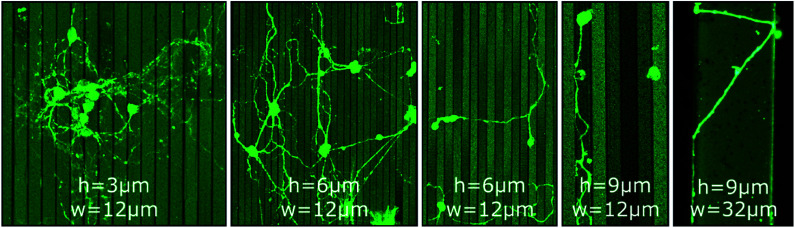
CLSM images of MCGCs at 10–11 DIV on steps of varied height (*h* = 3 μm, 6 μm and 9 μm) and width (*w* = 12 μm and 32 μm) produced by RIE. All substrates have been coated with ParC and PDL.

In contrast to GSL-produced steps, structures produced by a combination of RIE with subsequent UV-NIL reveal sharp, well defined edges with 90° angles. Thus, such structures are ideally suited to assess neurite pathfinding along competing growth options when exposed to barriers of different heights. Three different cases are observed on steps of 3 μm, 6 μm, and 9 μm height (Fig. 2). At 3 μm steps, the orientation of neurites in the network is statistically random with neurites freely extending over the steps. On 6 μm steps, neurites in networks have a predominant orientation along the edges of the steps and somata are mostly settled on lower steps and in pits. However, cross-linkage across the top steps is sufficient for network connections between the pits. Noteworthy, neurites of single or only a few neurons are switching between perpendicular and parallel orientation along the step edges. On 9 μm steps, no network formation across the steps is observed. Neurites are completely deflected when approaching a lower step edge, which leads to confinement in the pits and guidance along the step edge direction.

Li *et al.* reported a threshold height of 10–11 μm for cortical cell cultures in crossing and turning on steps coated with PDL.^51^ The authors explicitly noticed that the threshold is of the same magnitude as the growth cone dimension of cortical neurons. Hence, the results indicate that the size of the growth cone is also the defining factor for the threshold for MCGCs in crossing steps. When encountering a step of larger vertical dimensions than its dimension, the cone would need to turn in a 90° angle to cross onto the next step while turning in plane offer alternative pathways with smaller bending angles. Thus, neurites are entrapped by vertical walls of 9 μm. When encountering smaller steps, the bending angle needed to extend onto the next plane is reduced which in turn increases the likelihood of crossing.

The orientation of neurites perpendicular to 6 μm steps for low density cell cultures may be explained by the presence of topography as single guidance cues in the absence of other cells nearby. When the growth cone senses an edge, it can either align with the edge or continue its trajectory across the edge to sense for the next cue. The closest way to the next cue in form of the next step edge is directly 90° across the step. This form of perpendicular contact guidance has previously been observed on grooves of sub-micron dimensions and on radial neurite bundles.^52–54^

**Fig. 3:**
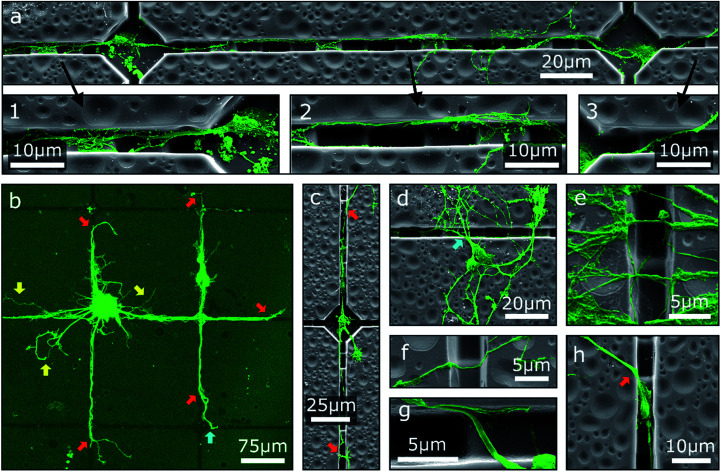
False-colored SEM images and a CLSM image of MCGCs on chessboard structures produced by GSL after 6 DIV. (a) Two connected neurons. Three magnified areas of interest show (1) guidance along the bottom of a channel, (2) guidance on the sidewall of a channel, and (3) a neurite pathway exiting a channel. (b) Red arrows indicate spots, where neurites leave the channels. Yellow arrows indicate neurites, that are not guided. Blue arrows indicate re-entering of neurites into channels. Neurites originating from a cell cluster and somata that are settled on top of channels. (c–h) Examples of neurites exiting, entering, and crossing channels.

One possibility to introduce an additional degree of order to neuronal networks cultivated on step structures is the addition of further topographical contact guidance. To this end, chessboard structures of cavities connected by channels with integrated steps produced by GSL were utilized as cell culture platforms for MCGCs. The height difference between consecutive steps is 2 μm as in the first scenario. Since neurites are trapped in the channel, we observe, in agreement with our previous results, no deflection at the steps because the angle of approaching the step is larger than 60°. Two neurons in adjacent cavities are shown in Fig. 3a. Neurites in the displayed channels show exemplary behavior for pathfinding observed on all of these investigated substrates. Specifically, neurites originating from cell somata that are settled in cavities are mostly extended towards the channels. Over a certain distance, the neurites are guided over the steps along the bottom of the channels (1). At some point, typically after crossing one of the top-level steps, the neurites attach to the channel wall and do not follow the steps back downwards (2) pointing to morphological guidance at the corner. Therefore, some neurites subsequently leave the channels. However, in the displayed case the main trajectory remains inside the channel and leads back down onto the channel bottom. In contrast, other neurites leave the channel after crossing the channel bottom towards the other edge when approaching a higher step level (3) indicating that the growth cone fumbles in a preferred forward direction.

The overall guidance effect of the channels is showcased in Fig. 3b. Most neurites originating from a cell cluster settled near a cavity as well as the neurites of somata settled on the top rim of a channel are extended into the channels. Guided neurites are apparently longer than neurites without contact guidance, which may indicate an acceleration of the neurite outgrowth through contact guidance in the channels similar to the effect shown for neurites inside of tubes.^17^ Nearly all observed neurites leave the channels at some point—predominantly at the highest step—and extend onto the substrate surface without 2.5D structuring. Note, observed neurites of somata settled inside of cavities typically exit the channels before reaching an adjacent cavity (Fig. 3c) indicating that the chosen distance between two cavities is too large to define connected neuronal networks when steps are introduced in the outgrowth path.

While neurite guidance along the channels is observed in the cases described above, a majority of the somata is unspecifically settled on the flat surface (Fig. 3d). Accidentally, single neurites from these somata are extended into the channels, but a large part of the neuronal networks is formed on the surface without modulation. Neurites of these networks, especially in case of neurite bundles, build suspended bridges over the channels (Fig. 3e) or extend down on one side wall and up on the other side wall without changing the trajectory (Fig. 3f and g). This observation again points to the fact of a preferred forward direction of the outgrowth by the growth cone. In a special case, some small somata are settled inside of a channel. Neurites can then be extended into the channels as well as onto the flat surface; no specific preference has been found in this case (Fig. 3h).

In intermediate summary, our observations show that steps and walls produced by GSL are sensed by the growth cone. The effect is not strong enough to reliably build ordered 2.5D networks on the structured surfaces, but some neuron caging and neurite guidance is given. The path chosen by neurites after encountering the border of a step or a channel is dependent on the angle of approach. At large angles, neurites tend to cross steps and channels without change of trajectory. At small angles, they tend to follow the step edge direction or channel. This behavior is analogous to stripe ‘tracking’, observed for neurites encountering stripes of fixed chemical guidance cues laminin and fibronectin.^55^ High probability of neurites exiting the channels at some point can be attributed to frequent directional changes of the trajectories induced by the step structure inside the channels. Neurites have previously been shown to cross boundaries more often on surfaces with multidirectional patterns than neurites on unidirectional patterns.^13^ The observation of longer neurites when extended through channels fits previous findings where axons on anisotropic patterns where significantly longer than axons on flat surfaces.^14^

### Chemical guidance by poly-d-lysine

**Fig. 4:**
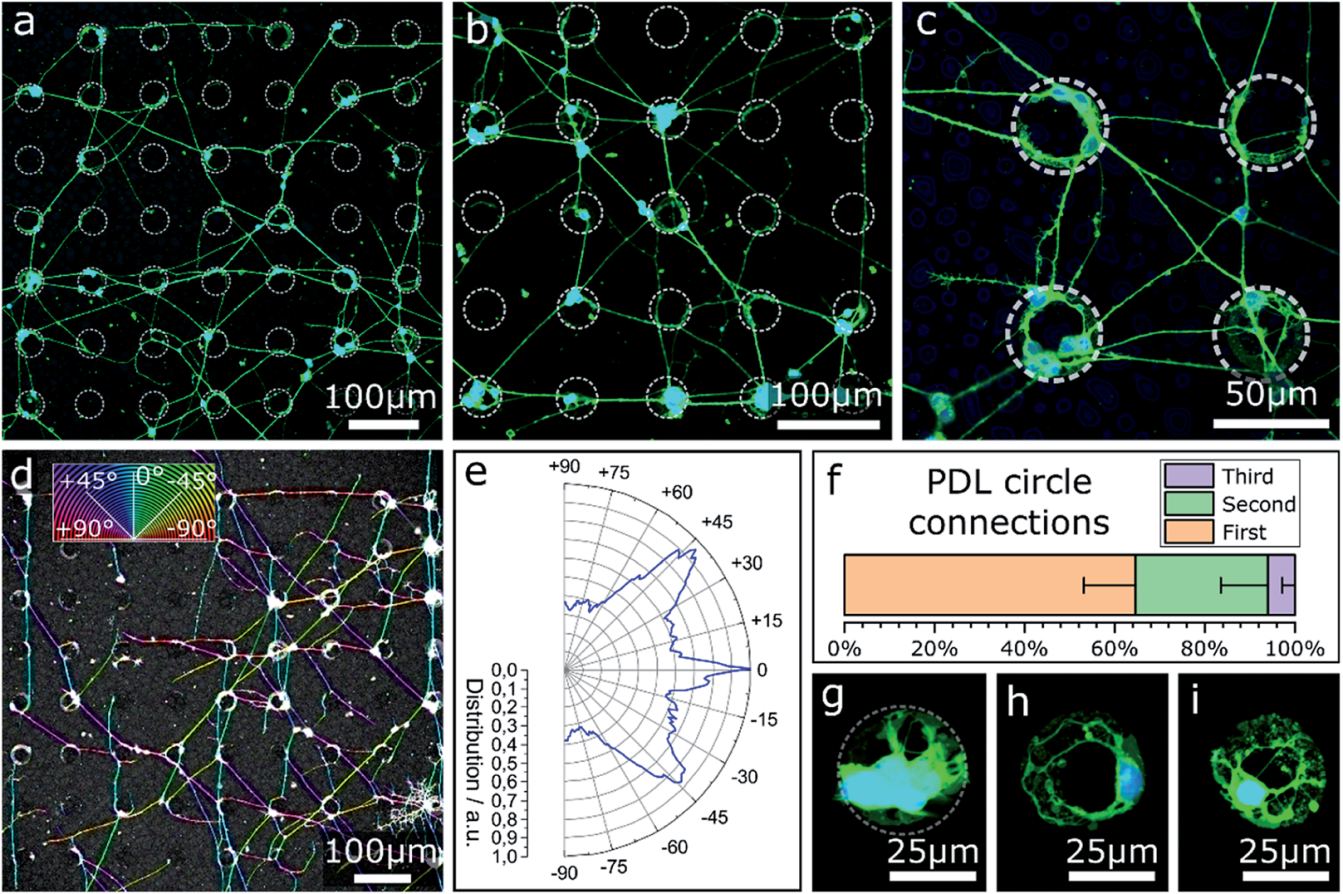
CLSM images and orientation analysis of neurons cultured on PDL circles with diameters of approx. 36 μm printed on an Al_2_O_3_ surface (7 DIV). (a–c) Circles printed in a pattern of 90 μm periodicity and an average distance between two circles of 54 μm. (d) Color-coded confocal image of neurons cultured on PDL circles in a pattern of 90 μm. Coloration of neurites corresponds to the neurite direction relative to the pattern. (e) Circular histogram of the normed distribution of neurite orientations on the pattern. (f) Percentage share of neurite pathways between nearest (first), second nearest and third-nearest circles. (g–i) Circles printed in a pattern of 190 μm and an average distance between two circles of 154 μm.

In the previous sections only contact guidance at steps and corners has been investigated. Note, the culture substrates have been uniformly coated by a PDL layer. However, selective adhesion coating can support contact guidance along the surface topography. The effect of selective surface coating with PDL on MCGCs was first tested on flat substrates without contact guidance to evaluate the impact as chemical guidance factor. The neurons were cultivated on glass substrates with an Al_2_O_3_ surface coating. Droplets of PDL solution were printed in a pattern of circles with approx. 36 μm diameter. Confocal microscope images of cultured cells for circles printed every 90 μm are shown in Fig. 4a–c. 89 ± 5% of all somata are settled directly on or are attached to circular areas covered with PDL. A network of neurites spans between the circles. In the rare case of somata that are located between circles, their neurites are directly extended to the nearest circles. Within the coated areas, a higher number of somata and neurites are attached and extended on the outer rim than in the center.

The results indicate a very strong PDL reliance of cell attachment and migration of somata on the substrate. Cell adhesion near the edges of circles indicates an uneven distribution of PDL residue in the rings, likely due to the so-called coffee-ring effect, known to occur in ink-jet printing of polymers.^56^ During neurite outgrowth, PDL clearly functions as a guidance cue. Orientational analysis of the neurite growth directions in case of PDL droplets with 90 μm periodicity (Fig. 4d and e) reveals that the simple quadratic pattern is sufficient to introduce a certain order to the network: dominant neurite orientation towards 0° and ±45°. Spreading of most neurites between nearest and second-nearest circles (Fig. 4f) results in preferred directions either parallel or diagonal to the printed pattern.

In contrast, Fig. 4g–i show close-ups of cultured neurons on/in circles printed with the same amount of PDL—same diameter—but every 190 μm where nearly all cellular material is attached at the PDL spots. Neurites of vital cells are exclusively extended inside the boundaries of the coated area. This result indicates that the minimal distance between two PDL circles of 154 μm (edge-to-edge) exceeds the maximal distance neurites of MCGCs span without attractive chemical guidance cues. When the distance between PDL-covered areas is too large, neurites retract and start sensing in a different direction. As a result, no neuronal networks can be formed over PDL coating with large distance spacing.

### Combination of contact and chemical guidance

**Fig. 5:**
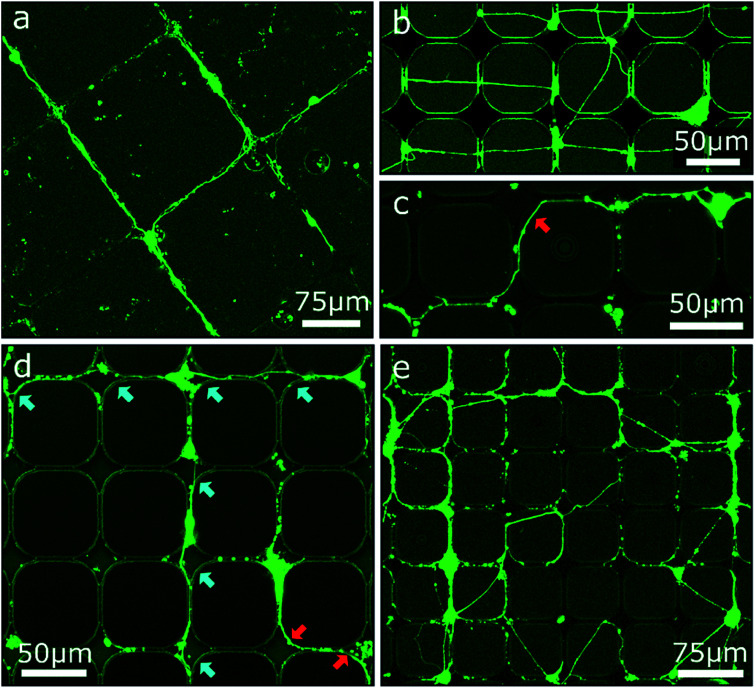
CLSM images of MCGCs at 6–8 DIV on chessboard structures produced by RIE and UV-NIL. (a) Cavities of 10 μm depth and steps in channels of 5 μm height coated with PDL with a materials printer. (b–e) Cavities of 14 μm depth and steps in channels of 4 μm height. PDL coated by a material printer (b) and with a micromanipulator (c–e). Red arrows indicate neurites guided at the top edge of cavities. Blue arrows indicate neurites at the bottom of cavities.

Based on the results of the first section, chessboard structures of cavities and channels with steps were produced by RIE as well as UV-NIL (see Fig. S2†) and were cultivated with MCGCs to obtain ordered 2.5D neuronal networks. But this time, PDL was not coated onto the entire surface, instead PDL was specifically and spatially applied to enhance the selectivity of cell adhesion only locally. First, PDL was coated with a materials printer targeted to the cavities. Note, it is reasonable to assume that droplets with PDL solution spread through the channels through capillary forces before evaporation of the solvent, leading to PDL coating that is homogenously distributed in the modulated area.^46^ Using this surface treatment, guidance in the confined area is achieved and a neuronal network spans through the channels and cavities (Fig. 5a). However, neuronal somata are mostly not settled in the cavities, but on top of the substrate's surface or in the channels themselves. Most likely, droplets of PDL solution aimed at the cavities were deposited onto the channels due to the insufficient accuracy of the stage alignment. As a result, somata are settled in the channels and neurites are spread on straight lines across the surface of the sample (Fig. 5b). Second, and in contrast, utilizing a pipette mounted at a micromanipulator to inject PDL into cavities and channels with the same design results in neuronal networks with somata exclusively attached to the cavities and with several neurites that spread through the channels and along the bottom of the cavities (Fig. 5c and d). Some neurites are partially guided along the top edges of the channels and cavities. When clusters of several cells are settled in cavities, some neurites are spread across the surface towards different cavities (Fig. 5e). This design was tested with step heights between 2 μm and 10 μm. Overall, neuronal networks in RIE-etched structures in combination with chemical cues have a high degree of order and can be used to successfully guide neuron outgrowth along 2.5D pathways for step heights of 4 μm and below. In accordance to the findings on step structures in the first section, step heights above 6 μm hinder neurite elongation; the observed neurite guidance in those cases occurs mostly for clusters along the top edge. Within this section we showed how the combination of chemical cues for guidance with contact guidance in channels can be employed to build neuronal networks in 2.5D. Our conclusive experiments revealed that spatially controlled chemical cue application, sharp edges, and step heights above 6 μm are needed for optimum guidance.

## Conclusion

We utilized grayscale lithography, reactive ion etching, and ultraviolet nanoimprint lithography to fabricate rapidly reproducible and cost-efficient cell culture platforms with well-defined 2.5D topography on the micron scale. Murine cerebellar granule cells were successfully cultivated on these substrates and formed low-density neuronal networks. Neurites were extended on 2.5D pathways. Vertical walls with certain height and sharp 90° edges confined neurite extension in a defined space on the bottom plane. The critical step height for neurite caging has been determined to be larger than 6 μm. However, every change in the trajectory raised the probability of neurites extending alongside walls and exiting the well-defined channels over the top edge. Selective chemical coating determines potential cell adhesion spots and areas of neurite outgrowth predominantly to the coated areas. Coated cavities act as efficient neuro-cages. Thus, with a combination of chemical and topographical guidance, ordered 2.5D neuronal networks have been successfully generated and maintained. Our results of guided neuronal outgrowth experiments on 2.5D structures prepared by semiconductor industry compatible techniques might pave the way to artificial microstructured cell culture substrates and might form the stepping stone for linking tailor-made semiconductor devices with neuronal cell cultures.

## Methods

### Grayscale lithography

Glass wafers were dehydrated at 200 °C for 10 min. AZ4562 photoresist (MicroResist) was deposited *via* spin coating at 2000 rpm for 30 s to get 10 μm thick layers and the wafers were kept on level ground at room temperature (RT) for 10 min. Optimal prebake time was 1 min at 60 °C and ramped up to 100 °C for 10 min. After GSL, the samples were developed in AZ826 MIF (metal ion free; MicroResist) for 3.5–6 min, depending on structure depth. Optical GSL was carried out using a DWL66+ laser writer (Heidelberg Instruments) equipped with a 363 nm Ar^+^ laser (max. 360 mW). Grayscale files were prepared with AutoCAD (AutoDesk).

### Reactive ion etching

Photomasks were produced with the DWL66+ laser writer using prefabricated photo mask blanks (G Materials) metalized with Cr and coated with 530 nm MICROPOSIT S1800 photoresist (MicroChem). The respective exposure patterns were prepared with AutoCAD. The wafers were heated to 115 °C for 50 s for postbake and developed in AZ826 MIF for 3.5 min. In preparation for RIE, Si wafers were spin coated with LOR5A at 4000 rpm and AZ4562 for 30 s at 4000 rpm. Prebake time was 1 min at 60 °C, ramped up to 100 °C for 6 min. Transferring the desired structures into the resist through contact lithography, the coated wafers were exposed for 15 s with 13 mW cm^−1^ of 365 nm UV light using an MJB4 mask aligner (Süss MicroTec). Substrates (silicon, 〈100〉 ±0.5°, Czochralski grown, SiegertWafer) were treated in a Si 500 inductively-coupled plasma (ICP)-RIE plasma etcher (Sentech). The gaseous mixture consisted of 50 standard cubic centimeters per minute (sccm) SF_6_ feed gas, 70 sccm C_4_F_8_ for sputtering and 5 sccm O_2_ for glow discharge. The stage temperature was set to 0 °C, ICP power was set to 400 W and radio frequency was set to 15 Hz with a chamber pressure of 1.02 bar. Subsequently, photoresist was removed with acetone in an ultrasonic bath.

### UV nanoimprint lithography

UV-NIL was utilized for the duplication of structures previously produced by RIE. These structures served as master stamps for the UV-NIL production. The master stamps were covered with a layer of fluorosilane (1*H*,1*H*,2*H*,2*H*-perfluorodecyltrichlorosilane, 96%, Alfa Aesar) as anti-adhesive coating by CVD. The stamps were first cleansed by sonication in acetone and isopropyl alcohol (IPA), followed by 2 min of ozone exposure in a UV/ozone cleaner (UVO Cleaner Model 144AX Series, Jelight Company). Following ozone treatment, CVD of fluorosilane was performed in a Vacutherm vacuum oven (Fisher Scientific) for 30 min at 80 °C and 0.4 bar. Common glass coverslips (20 mm, #1, CarlRoth) were utilized as resin substrates. The substrates were cleansed and exposed to ozone plasma for dehydration analogous to the stamps. OrmoPrime® 08 (MicroResist) was applied as adhesion layer through spin coating for 1 min at 4000 rpm and hardbaked on a hotplate for 5 min at 150 °C. OrmoStamp (MicroResist), a polymer with glass-like properties and high UV-NIL resolution, was used as resist. Single droplets were positioned centrally on the stamps, before glass substrates were placed on top without additional pressure. UV-curing was performed in an MJB4 mask aligner for 2 min with 13 mW cm^−1^ of 365 nm UV light. The resulting negatives of the original master stamps function as replica stamps in a second UV-NIL step with identical parameters, which produces the substrates utilized as cell culturing platforms.

### Surface coatings

#### Alumina

Al_2_O_3_ was deposited by ALD utilizing trimethylaluminum [Al(CH_3_)_3_] and water as precursors in a custom-built ALD system. On planar control substrates, a layer thickness of 15 nm was determined by spectral ellipsometry.

#### Parylene C

ParC was coated on cell culture substrates by CVD in a SCS Lab-coater® 2 (Speciality Coating Systems). In previous experiments with 0.7 g ParC all surfaces of structures, including interior surfaces of microtubes, were coated with a layer of approx. 130 nm thickness.^20^ For this work, 0.5 g were used for coating.

#### Poly-d-lysine

Regardless of the PDL deposition method, all substrates were treated with ozone plasma at 3.5–5 W for 3–5 s in a plasma system (Femto, Diener Electronic) in advance to increase the hydrophilicity of the surface.

For nonspecific PDL coating onto the entire surface of the culture platform a droplet of approx. 100 μl cm^−2^ PDL solution (0.01 g l^−1^, mol wt 30–70k, Sigma Aldrich) was pipetted onto the substrates. Resting time was at least 30 min. After removing the PDL solution, the substrates were cleansed with DI water (18.2 MΩ cm) two or three times to remove excess PDL molecules.

Patterns of PDL were printed utilizing a Dimatix Materials printer DMP-2831 (Fujifilm) with 16 nozzle cartridges that eject 1 pl droplets of liquid. Piezo-element voltage was set to 20–25 V, with a jetting frequency of 2.5 kHz. The temperature of the nozzle chamber as well as the sample holder was set to 21 °C. The distance between write head and substrate was set to 600 μm. The viscosity of PDL solution, estimated based on droplet velocity and piezo-element voltage, is approximately 13 cP.

A custom-built upright patch clamp setup was utilized for site-specific PDL coating in cavities and grooves. The setup consists of a microscope, an objective with a large working distance, a micromanipulator and freshly prepared glass pipettes with openings of down to <1 μm.

#### Laminin

Right before cultivation, all substrates were additionally coated with laminin by incubation with laminin solution (10 μg ml^−1^) for 20 min.

### Cell culture

The substrates were cultivated with wild type murine cerebellar granule cells. All mice were 6–7 days old at the time of extraction. A detailed description of the isolation process has been previously given by Loers *et al.*^57^ Culture medium was Neurobasal A (Thermo Fisher Scientific) supplemented with penicillin/streptomycin (1%), bovine serum albumin (1%), insulin (10 μg ml^−1^), l-thyroxine (4 nM), transferrin holo (100 μg ml^−1^), sodium selenite (30 nM) (all Sigma Aldrich), fetal bovine serum (6%, Capricorn Scientific – FBS Advanced) and Gibco B-27 (50×, Thermo Fisher Scientific). Cell concentration was adjusted to 2 × 10^5^ cells per ml. Cells were plated in droplets of approx. 100 μl cm^−2^ suspension and left to settle for 1 h at 37 °C and 5% CO_2_ in the incubator. Afterwards, 3 ml cell medium was added per 35 mm Petri dish (Fisher Scientific, Biolite). Cytosine arabinoside (3 μM) was added after 24 h. Medium was renewed after 2 DIV and subsequently every 2–3 days.

### Animals

C57BL/6 mice were bred at the animal facility of the University Medical Center Hamburg-Eppendorf and maintained at 22 °C on a 12 h light/12 h dark cycle and provided with food and water *ad libitum*. All experiments were conducted with mice of either sex and in accordance with the German and European Community laws on protection of experimental animals. Procedures used were approved by the responsible committee of The State of Hamburg (permission no. Org_679). Experiments were carried out and the manuscript was prepared following the ARRIVE guidelines for animal research.

### Confocal laser scanning microscopy

For CLSM, neurons were stained using Neurite Outgrowth Staining Kit (Invitrogen, Thermo Fisher). Growth medium was removed and the substrates were treated with 1× dye mixture in Hank's Balanced Salt Solution (HBSS) or Dulbecco's Phosphate-Buffered Saline (DPBS) for 15 min at 37 °C and 5% CO_2_. Imaging was performed with a Leica TCS SP8 confocal microscope (excitation at 488 nm and 552 nm wavelength; detection between 494–530 nm and 558–610 nm). Rendering of images to 2D stacks was performed using Leica LAS X software or the Fuji distribution of ImageJ.^58,59^ The OrientationJ plugin in ImageJ was used for directional analysis of neurite orientation.^60,61^

### Scanning electron microscopy

In preparation for SEM, cells were fixated and dried by exposure to paraformaldehyde in HBSS (4%) for 20 min at 37 °C. Substrates were rinsed three times with purified water and successively submerged in 10%, 25%, 50%, 75% and 99.5% v/v ethanol absolute (VWR Chemicals) solution for 10 min each. Subsequently, the substrates were either air dried or critically point dried for optimal preservation (Autosamdri-815 Series A, Tousimis). Substrates were stored under vacuum in a desiccator until further use. All substrates were coated with approx. 30 nm Au by sputter coating (K550 Emitech Sputter Coater) before SEM imaging (Zeiss Crossbeam 550).

## Author contributions

C. F. and L. R. produced the substrates and conducted the analysis of the culturing results. C. F. and J. H. cultivated the neurons. G. L. prepared the murine cerebella. C. F., J. H. and R. Z. wrote the manuscript. C. F and R. B. conceived the study. R. Z. and R. B. supervised the project. R. B. assisted in manuscript finalization. All authors significantly contributed to the scientific discussion during data evaluation and manuscript preparation. All authors conducted a final proof reading.

## Conflicts of interest

There are no conflicts to declare.

## Supplementary Material

NA-002-D0NA00549E-s001
