# Supplementary material for: Neurite guidance and neuro-caging on steps and grooves in 2.5 dimensions
Source: Nanoscale Adv. 2020 Jul 14;2(11):5192–200. doi: 10.1039/d0na00549e (PMC9417336; doi:10.1039/d0na00549e)
Supplement: NA-002-D0NA00549E-s001 [file NA-002-D0NA00549E-s001.pdf]

Electronic Supplementary Material (ESI) for Nanoscale Advances.
This journal is © The Royal Society of Chemistry 2020

Supporting material: Neurite guidance and neuro-caging on steps and grooves in 2.5 dimensions

Cornelius Fendler[a], Jann Harberts[a], Lars Rafeldt[a], Gabriele Loers[b], Robert Zierold[a*], Robert H. Blick,[a,c]

[a]*Center for Hybrid Nanostructures (CHyN), Universität Hamburg, 22761 Hamburg, Germany*
[b]*Center for Molecular Neurobiology Hamburg (ZMNH), University Medical Center Hamburg-Eppendorf (UKE), Hamburg 20251, Germany.*
[c]*Material Science and Engineering, College of Engineering, University of Wisconsin-Madison, Madison, Wisconsin 53706, USA.*

Substrates produced by grayscale lithography (GSL)

Cross sections of step structures in AZ4562 as result of exposure tests at 22 mW laser power are shown in Figure S1 a. Since photon absorption in the resist is a nonlinear process, exposure with linear grading leads to an exponential decrease of step height towards larger depths. If the goal is the production of complex geometries, results of an intensity test can be fed into computational calculation to convert desired features into fitting gray scale values. Correct values for simple structures without the demand for nanoscale accuracy, such as the ones described in this chapter, can be directly estimated based on the test results. An exemplary step structure created by GSL with a targeted step height of 2 µm is shown in Figure S1 b. The gray values were set to 42, 122 and 228 to reach 2 µm, 4 µm and 6 µm depths. A profile corresponding to such a step structure is shown in Figure S1 c. The profile indicates slightly rounded edges. Figure S1 d shows examples of a chessboard structures produced with GSL. The design connects cavities through channels with integrated steps of 2 µm height with each other.

Substrates produced by reactive ion etching (RIE)

A plot of RIE etch depth in dependence to process time is shown in Figure S2 a. Slight variations in the etch rate can be attributed to temperature fluctuations in the reaction chamber. The etch rate determined by linear regression of the etch depths is (9.8 ± 0.1) nm/s. To obtain multiple steps in one structure, up to three consecutive cycles of contact lithography and RIE were executed. An example of a structure resulting from two cycles is shown in Figure S2 b. Etching of channels with 10 µm gaps every 40 µm in one and overlaying continuous channels in the other cycle results in steps with the height defined by RIE in the cycle with the gaps included in the design. Figure S2 c shows multiple substrates for which the described principle was utilized to produce identical boundaries of 10 µm height defining channels that connect cavities with 2 µm – 10 µm additional depth, resulting in distinct steps at the channel entrances/exits. Isolated cavities were etched first, followed by the design including the channels. Steep sidewalls form 90 ° corners at the bottom of the structures. Slight misalignment of the exposure designs and interference patterns are visible due to limited accuracy of contact lithography with mask aligners during the second RIE circle. At high overall depth of 18 µm and 20 µm, deviations from the design increase with residues and uneven surface inside the cavities.

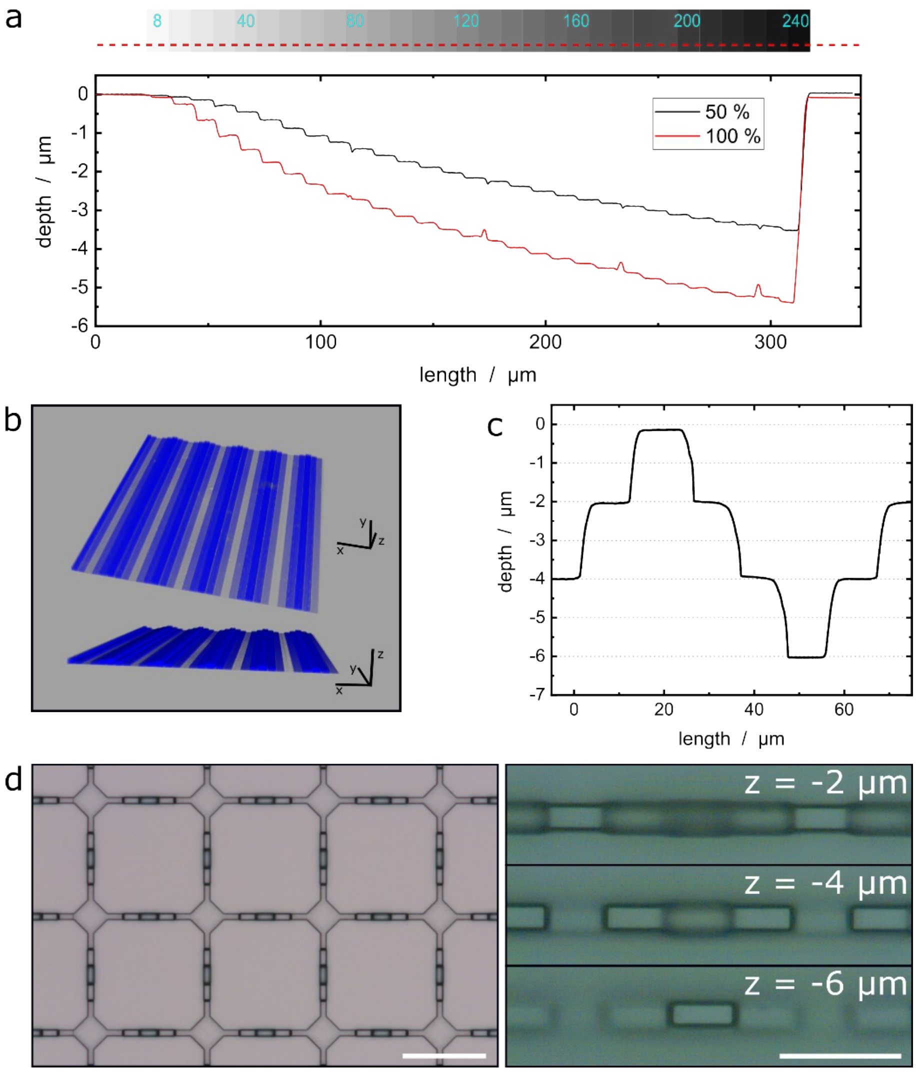

Figure S 1: Profiles and images of structures produced by GSL in AZ4562 photoresist with 22 mW laser power. The profiles were measured with a DektakXT profilometer (Bruker). (a) Results of exposure tests. A 50 % filter was installed in the beam path of the laser for the black plot in contrast to no filter for the red plot. The gray bars at the top give a top view onto the gray values of the exposure design. From left to right, the gray values increase in discrete steps of 8, which linearly correlate with the local UV laser intensity during the writing process. The red dashed line represents the profiles displayed in the plot. (b) CLSM 3D stack of the step structure. Scale: 40 µm. (c) Profile of a step structure created by exposure with alternating gray values of 0, 42, 122 and 228. (d) Brightfield images of a chessboard structure produced with the same gray values of 0, 42, 122 and 228. Cavities are connected through channels with steps. The focus of the right images is shifted by -2 µm top to bottom. Scale bars: 50 µm, 20 µm.

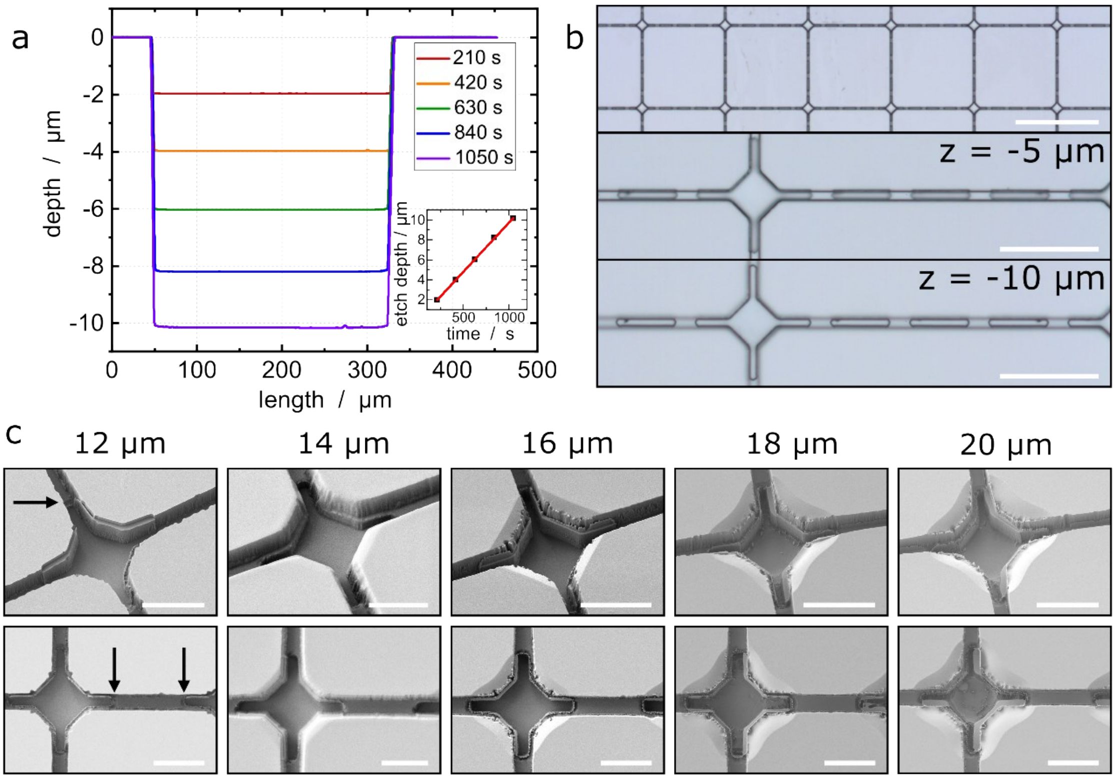

Figure S2: Profiles and images of structures produced by RIE in silicon. (a) Cross section profiles of grooves measured with a profilometer. A plot of the etch depth against time is shown in the small graph. The etch rate is (9.8 ± 0.1) nm/s. (b) Optical images of a chessboard pattern created by two consecutive etch steps. Cavities are connected through channels with steps. The focus is set to -5 µm in the middle and -10 µm in the bottom image relative to the silicon surface. Scale bars: 200 µm (top), 50 µm (middle and bottom). (c) SEM images of chessboard structures with isolated cavities with increasing depth from 12 µm to 20 µm from left to right etched in a first step and cavities connected by continuous channels with 10 µm depth edged in a second step, resulting in steps at the cavity exits accordingly (indicated for 12 µm cavities with arrows). Scale bars: 20 µm.
